# Photobiomodulation repairs the blood–spinal cord barrier in a mouse model of spinal cord injury

**DOI:** 10.4103/NRR.NRR-D-24-01098

**Published:** 2025-03-25

**Authors:** Yangguang Ma, Yi Liu, Dongsheng Pan, Jiawei Zhang, Zhuowen Liang, Yi Wang, Xueyu Hu, Zhe Wang, Tan Ding

**Affiliations:** 1 Department of Orthopedics, Xijing Hospital, Fourth Military Medical University, Xi’an, Shaanxi Province, China; 2 Shaanxi Shuoguang Qifu Medical Technology Co., Ltd., Xianyang, Shaanxi Province, China

**Keywords:** autophagy, blood–spinal cord barrier, endothelial cell, mitochondria, neuroinflammatory, photobiomodulation, PTEN-induced kinase 1, repair, spinal cord injury, tight junction

## Abstract

The blood–spinal cord barrier is crucial for preserving homeostasis of the central nervous system. After spinal cord injury, autophagic flux within endothelial cells is disrupted, compromising the integrity of the blood–spinal cord barrier. This disruption facilitates extensive infiltration of inflammatory cells, resulting in exacerbated neuroinflammatory responses, neuronal death, and impaired neuronal regeneration. Previous research has demonstrated that photobiomodulation promotes the regeneration of damaged nerves following spinal cord injury by inhibiting the recruitment of inflammatory cells to the injured site and restoring neuronal mitochondrial function. However, the precise mechanisms by which photobiomodulation regulates neuroinflammation remain incompletely elucidated. In this study, we established a mouse model of spinal cord injury and assessed the effects of photobiomodulation treatment. Photobiomodulation effectively cleared damaged mitochondria from endothelial cells in mice, promoting recovery of hindlimb motor function. Using microvascular endothelial bEnd.3 cells subjected to oxygen–glucose deprivation, we found that the effects of photobiomodulation were mediated through activation of the PINK1/Parkin pathway. Additionally, photobiomodulation reduced mitochondrial oxidative stress levels and increased the expression of tight junction proteins within the blood–spinal cord barrier. Our findings suggest that photobiomodulation activates mitochondrial autophagy in endothelial cells through the PINK1/Parkin pathway, thereby promoting repair of the blood–spinal cord barrier following spinal cord injury.

## Graphical abstract

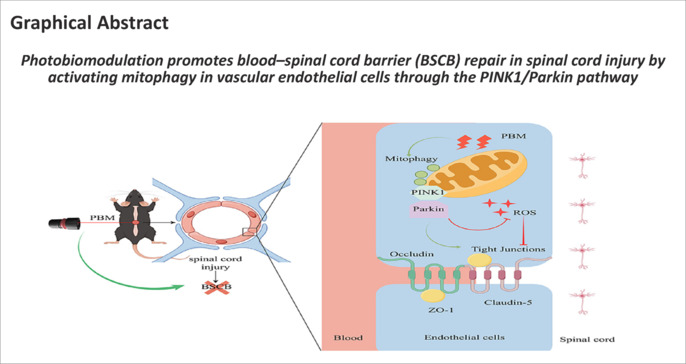

## Introduction

Spinal cord injury (SCI) is caused by events such as car accidents, violence, and falls from heights, and results in motor and sensory nerve dysfunction (Alizadeh et al., 2019; Zheng and Tuszynski, 2023). Patients with SCI often suffer from serious complications such as lung infections, pressure sores, anxiety, depression, and suicidal tendencies. Approximately 130,000 new cases of acute SCI are estimated to occur worldwide each year (Chariker et al., 2019; Cragg et al., 2019). Owing to the lack of effective treatment methods, the clinical prognosis is often poor (Zhang et al., 2022). Efforts to develop new, safe, and effective treatment strategies to promote nerve tissue repair and functional restoration are key in the field of SCI research.

The pathological process of SCI can be divided into two stages: primary injury and secondary injury. The primary injury is caused by external mechanical forces that directly act on the spinal cord, resulting in direct damage to cells, blood vessels, and the blood–spinal cord barrier (BSCB) (Yao et al., 2018; Zhang et al., 2019). This damage is usually irreversible (Wu et al., 2021b). The secondary injury develops as a result of the primary injury, whereby disruption of the BSCB allows inflammatory cells such as microglia, macrophages, and neutrophils to invade the injured site, exacerbating local neuroinflammation and further hindering nerve regeneration and functional recovery (Brennan et al., 2016; Zhou et al., 2016). Therefore, restoring the integrity of the BSCB is an important goal for potential SCI treatments.

The BSCB is composed of a continuous layer of endothelial cells connected by tight junction (TJ) proteins, surrounded by astrocytes, pericytes, and microglial cells, and is essential for maintaining spinal cord homeostasis and neurological function (Abbott et al., 2010; Mizee and de Vries, 2013; Lee et al., 2019). TJ proteins mainly comprise proteins such as occludin, claudins, and zonula occludens (ZO) proteins, as well as adhesion molecules (Zihni et al., 2016; Kuo et al., 2022). The ischemic and hypoxic microenvironment resulting from SCI may cause an increase in endothelial cell apoptosis and a decrease in intercellular TJ protein expression (Kumar et al., 2018; Luo et al., 2022). Increasing the expression of intercellular TJ proteins and reducing endothelial cell apoptosis to restore BSCB integrity after SCI are effective strategies for reducing inflammatory cell infiltration and promoting nerve regeneration.

Mitophagy is a critical autophagy process that maintains cellular health by removing damaged mitochondria and promoting the elimination of mitochondrial deposits, and has antioxidant and anti-apoptotic effects (Ajoolabady et al., 2022; Liu et al., 2023). Mitophagy plays a pivotal role in preventing vascular endothelial cell apoptosis, thereby safeguarding the BSCB from injury (Chu et al., 2024; Xu et al., 2024). In SCI, the disruption of autophagic flux in endothelial cells leads to degradation of TJ proteins, ultimately resulting in BSCB damage (Zhou et al., 2016; He et al., 2023). During the ischemia–reperfusion phase that occurs after SCI, mitophagy regulates mitochondrial homeostasis and promotes cell survival (Wu et al., 2021a; Nie et al., 2022). The PTEN-induced kinase 1 (PINK1)/Parkin pathway is a prototypical mitophagy pathway, in which PINK1 primarily recruits Parkin to damaged mitochondria (Li et al., 2023a). This leads to ubiquitination of target proteins on the mitochondrial outer membrane, further inducing autophagy and degradation (Nguyen et al., 2016). PINK1 selectively accumulates in damaged mitochondria, recruiting Parkin to induce their degradation, thereby alleviating neuronal cell damage after SCI (Huang et al., 2020; Meng et al., 2022). For this reason, PINK1/Parkin-mediated mitophagy may play a crucial role in the treatment of SCIs.

Photobiomodulation (PBM), also known as low-level laser therapy, is a technique that uses low-level lasers in the infrared and near-infrared spectrum to improve the function of cells, tissues, and organs (Mussttaf et al., 2019; Song et al., 2024). PBM is believed to exert its effect mechanistically through cytochrome c oxidase in mitochondria: after absorbing red or near-infrared light, cytochrome c oxidase increases electron transfer, mitochondrial metabolism, and adenosine triphosphate production through photodissociation (Kuffler, 2016). Previous studies have confirmed that sustained PBM therapy can inhibit recruitment of inflammatory macrophages/microglia to the SCI site and promote neuronal survival in the injured area in rodents through the mitochondrial pathway (Ma et al., 2022; Zhu et al., 2022). However, the specific mechanisms by which PBM regulates the inflammatory microenvironment of the injury site and its impact on BSCB integrity after SCI are not fully understood.

Given the core roles of BSCB and mitophagy in regulation of the inflammatory microenvironment and neuronal survival after SCI, in this study we treated a crush-induced mouse model of SCI with PBM and explored the effects on BSCB repair, endothelial cell mitophagy, intercellular TJ protein expression, and motor function recovery. We also investigated the potential mechanisms by which PBM regulates TJ proteins between endothelial cells and the possible involvement of the PINK1/Parkin pathway.

## Methods

### Animals

In view of the fact that there are far more male than female patients with SCI, as well as the potential protective effect of estrogen against SCI (Shvetcov et al., 2023; Zhou et al., 2024), only male mice were used in this study. Male C57BL/6 mice (specific-pathogen–free), aged 6–8 weeks, weighing approximately 18–20 g, were obtained from the Animal Experimental Center of the Fourth Military Medical University, Xi’an, Shaanxi, China (license No. SYXK (Shaan) 2024-003). The animals were kept in clean, warm cages maintained at 22–25°C with a 12/12-hour light/dark cycle and had access to sufficient food and water. The animal experiments were approved the Ethics Committee of the Fourth Military Medical University (approval No. IACUC-20241420; January 1, 2024) and were performed in accordance with the Guide for the Care and Use of Laboratory Animals (National Research Council, 2011).

### Spinal cord injury model establishment

The mice were subjected to SCI using a standard bilateral spinal cord compression injury model, as we described previously (Ma et al., 2022; Ju et al., 2023). Injection with 0.6% pentobarbital sodium (10 mL/kg, Sigma-Aldrich, St. Louis, MO, USA) was used to anesthetize the mice. The spinal cord at T9 segment was fully exposed, modified Dumont series forceps (Fine Scientific Tools, Heidelberg, Germany) were placed vertically on both sides of the spinal cord, the forceps were clamped, leaving a gap of 0.3 mm, and the clamping of the spinal cord was maintained for 30 seconds. After clamping, the distal end of a disposable medical optical fiber (SGQF-GX-A/600, Shuoguang Qifu Medical Technology Co., Ltd., Xianyang, Shaanxi, China) was securely fixed to the spine and soft tissue at the T8 thoracic vertebra using absorbable sutures. The skin was disinfected with iodine, and the muscle layer was sutured together. Mice in the Sham group underwent the same procedure to expose the spinal cord tissue but were not subjected to spinal cord clamping. Immediately following surgery, each mouse was placed in a warm, enclosed environment until it regained consciousness. Bladder massage was performed twice daily to prevent urinary tract infections. Mice with lower limb edema or wound infection were excluded from the study.

### Photobiomodulation therapy

The mice subjected to SCI were randomly divided into the SCI + PBM treatment group and the SCI alone group, with 40 mice in each group. PBM treatment involved the use of a semiconductor laser therapeutic instrument (SGQF-JSJ-808-1, Shuoguang Qifu Medical Technology Co., Ltd.) and a disposable medical optical fiber. The safety and irradiation parameters of the optical fiber were previously validated in a pig model (Zuo et al., 2022). The optical fiber (cylindrical, diameter of 600 μm) was coated with highly transparent medical-grade silica to ensure its flexibility and biocompatibility while maintaining its optical performance. A calibrated optical sensor was used to confirm that the output power of the fiber matched the specified power settings. For 50 minutes each day, the SCI area of the mice was irradiated at a power density of 50 mW/cm^2^. The experimental timeline is shown in **Additional Figure 1**.

**Figure 1:**
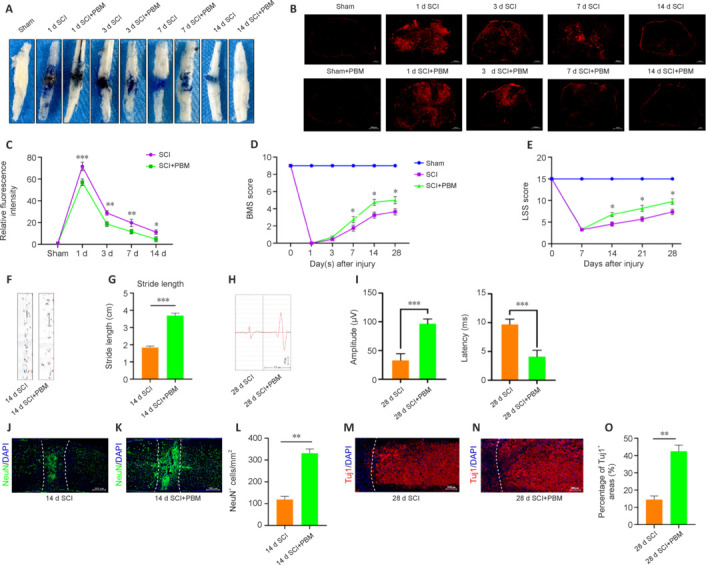
PBM restores BSCB integrity and promotes spinal cord injury repair. (A) Representative images of the spinal cord showing EB extravasation in Sham, SCI, and SCI + PBM mice at 1, 3, 7, and 14 days after injury (*n* = 6 animals per group). EB extravasation peaked at 1 dpi. At 1, 3, 7, and 14 dpi, PBM reduced EB extravasation. (B) Representative immunofluorescence images showing EB extravasation in coronal sections of the spinal cord in the injured area of mice in the Sham, SCI, and SCI + PBM groups at 1, 3, 7, and 14 days after injury (*n* = 6 animals per group). At 1, 3, 7 and 14 dpi, PBM reduced EB extravasation. Scale bars: 200 μm. (C) Quantitative analysis of EB fluorescence intensity (*n* = 6 animals per group). (D) BMS scores of mice in the Sham, SCI, and SCI + PBM groups at different time points after SCI (*n* = 12 animals per group). (E) Louisville Swimming Scale (LSS) scores of mice in the Sham group, SCI group, and SCI + PBM group at different time points after SCI (*n* = 6 animals per group). (F, G) Representative footprint analysis images of mice in the SCI and SCI + PBM groups on day 14 after SCI (*n* = 12 animals per group). The step length of mice in the SCI + PBM group was significantly longer than that in the SCI group. Red: forelimbs; blue: hindlimbs. (H) Representative neurophysiological images from each group, recorded 28 days after SCI. (I) Amplitude and latency measurement of MEPs in each group of mice. (J–L) Immunofluorescence staining of NeuN^+^ (green, Alexa Fluor 488) neurons at the spinal cord injury site of mice in the SCI and SCI + PBM groups on day 14 post-injury, with the dashed line showing the injury margin (*n* = 6 animals per group). There were more NeuN^+^ neurons at the SCI site of mice in the SCI + PBM group than in the SCI group. Scale bars: 200 μm. (M–O) Immunofluorescence staining of Tuj1^+^ (red, Alexa Fluor 594) neuron axons at the spinal cord injury site of mice in the SCI and SCI + PBM groups on day 28 after injury. Significantly more Tuj1^+^ axons were observed at the SCI site of mice in the PBM group than in the SCI group. The dotted line indicates the injury margin (*n* = 6 animals per group). Scale bars: 200 μm. Data are shown as mean ± SD. *P < 0.05, **P < 0.01, ***P < 0.001 (two-way repeated measure analysis of variance followed by Tukey’s *post hoc* test [C–E] or unpaired two-tailed Student’s *t*-test [G, I, L, O]). BMS: Basso Mouse Scale; DAPI: 4′,6-diamidino-2-phenylindole; EB: Evans Blue; PBM: photobiomodulation; SCI: spinal cord injury.

### Blood–spinal cord barrier permeability assessment

BSCB integrity was assessed by evaluating extravasation of Evans Blue (EB) dye. Mice were anesthetized by intraperitoneal injection of 0.6% sodium pentobarbital (10 mL/kg), then injected with EB solution (2%, Aladdin, Shanghai, China) via the tail vein. One hour later, the mice were euthanized by cardiac perfusion with saline and 4% paraformaldehyde (PFA). A 10-mm section of the spinal cord at the T9 level was removed and sliced into 10-μm sections using a cryostat (CM1900, Leica Microsystems, Wetzlar, Germany). EB fluorescence was observed using a Leica fluorescence microscope (SP8) and quantified using ImageJ software (v1.4.67, National Institutes of Health, Bethesda, MD, USA) (Schneider et al., 2012).

### Motor function assessment

The Basso Mouse Scale (BMS) (Basso et al., 2006) was used to evaluate recovery of hindlimb function in the mice. After pre-training, each mouse was placed individually in an open field for evaluation. The BMS was used to assess hindlimb joint mobility, trunk stability and balance, coordination between the forelimbs and the hindlimbs, paw position, toe spread, and tail position. Observation was conducted by two blinded independent investigators. Scores ranging from 0 to 9, representing complete paralysis to normal movement, were recorded before and 1, 3, 7, 14, and 28 days after surgery.

The Louisville Swim Scale was used to assess motor function recovery following SCI (Smith et al., 2006). Mice were placed in a water-filled tank and trained to swim from one side to the other. The Louisville Swim Scale was used to record and evaluate forelimb dependency, hindlimb movement and alternation, body angle, and trunk stability. Each mouse was tested twice, and the average score from the two tests was taken as the final score.

Footprint analysis was performed to analyze gait in mice in the SCI alone and SCI + PBM groups. This technique involves painting the forelimbs and hindlimbs with red and blue dye to record walking patterns, analyzing stride length and width at constant speeds (Isvoranu et al., 2021).

### Neuroelectrophysiology

Motor-evoked potentials were detected using the BL-422I Data Acquisition and Analysis System (TECH-MAN SOFT, Chengdu, Sichuan, China). On the 28^th^ day after injury, and a positive stimulation electrode was placed on the skull surface over the motor area of the mouse cerebral cortex, and a negative stimulation electrode was placed near the orbital bone. The recording electrode was inserted into the left or right gastrocnemius muscle, the control electrode was inserted into the distal tendon of the hindlimb muscle, and the ground electrode was placed under the skin on the back. The mice were stimulated with a 3-mA single square wave (2 Hz) for 0.2 ms, and motor-evoked potentials were recorded. Neurological function recovery was evaluated by calculating the time from the starting point of the first response wave to the highest peak and the amplitude of the motor-evoked potential of the mouse hindlimbs.

### RNA transcriptome sequencing and analysis

RNA transcriptome sequencing was used to compare the SCI and SCI + PBM groups at 14 days post-injury (dpi; *n* = 4 mice per group). Total RNA was isolated from spinal cord tissue samples using an RNAmini kit (Qiagen, Hilden, Germany) according to the manufacturer’s instructions. RNA quality was assessed by gel electrophoresis and Qubit (Thermo, Waltham, MA, USA). Strand-specific libraries were constructed using a TruSeq RNA Sample Preparation Kit (Illumina, San Diego, CA, USA) and sequenced by Genergy Biotechnology Co. Ltd. (Shanghai, China). The raw data were processed using Skewer (v0.2.2, https://github.com/relipmoc/skewer), and the data quality was assessed using FastQC (v0.11.2, https://www.bioinformatics.babraham.ac.uk/projects/fastqc/). DESeq2 software (v1.16.1, https://basespace.illumina.com/home/index) was used to identify known genes with differential levels of expression between the two sample groups, and differentially expressed genes (DEGs) between the two groups were defined as those with a *P*-value < 0.05 and a fold change in expression of greater than or equal to two. Then, Gene Ontology (GO; http://geneontology.org/; Ashburner et al., 2000) and Kyoto Encyclopedia of Genes and Genomes (KEGG; https://www.kegg.jp/) analyses were performed to determine enriched functions and signaling pathways among the DEGs. Significantly enriched pathways were defined as those with a *P*-value < 0.05 that included at least two DEGs.

### Cell culture and oxygen–glucose deprivation

Mouse brain microvascular endothelial cells (bEnd.3 cells, American Type Culture Collection, Manassas, VA, USA, Cat# CRL‐2299, RRID: CVCL_0170) were cultured in Dulbecco’s modified Eagle’s medium (Gibco, Tulsa, OK, USA) supplemented with 10% fetal bovine serum (Biological Industries, Kibbutz Beit-Haemek, Israel) and 1% penicillin-streptomycin (New Cell Molecular Biotechnology Co., Ltd., Suzhou, Jiangsu, China) at 37°C in a 5% CO_2_ incubator. The cells were passaged every 2–3 days until they reached 80%–90% confluence. Then, the bEnd.3 cells were washed with glucose-free, deoxygenated Dulbecco’s modified Eagle’s medium and incubated in a hypoxia chamber (Thermo Fisher Scientific, Waltham, MA, USA) supplied with a gas mixture of 5% CO_2_ and 95% N_2_ at 37°C for 12 hours. Control cells were cultured in Dulbecco’s modified Eagle’s medium containing 10% fetal bovine serum for the same amount of time. Cells were pretreated with the mitophagy inhibitor Mdivi-1 (1 μM, MedChem Express, Monmouth Junction, NJ, USA) for 2 hours prior to exposure to oxygen–glucose deprivation (OGD) and PBM (808 nm, 6 mW/cm^2^, 7 min/time, twice a day).

### Real-time polymerase chain reaction

Total RNA was extracted from cells or spinal cord tissues using a total RNA extraction kit (Omega Bio-tek, Norcross, GA, USA) according to the manufacturer’s instructions. Reverse transcription was performed using PrimeScript RT Master Mix (Takara Bio, Inc., Kusatsu, Shiga, Japan) according to the manufacturer’s instructions. The resulting complementary DNA was diluted 10-fold with RNase-Free ddH_2_O and stored at –20°C. The relative expression levels of the target mRNAs were normalized to glyceraldehyde-3-phosphate dehydrogenase (GAPDH) expression using the modified 2^–ΔΔct^ method (Livak and Schmittgen, 2001). The primer sequences are listed in **Table 1**.

**Table 1:** Information of primer sequences

Gene	Forward primer sequence (5'–3')	Reverse primer sequence (5'–3')
*ZO-1*	GAT AGT TTG GCA GCA AGA GAT GGT A	AGG TCA GGG ACG TTC AGT AAG GTA G
*Occludin*	CCT TCT GCT TCA TCG CTT CCT TA	CGT CGG GTT CAC TCC CAT TAT
*Claudin-5*	AGT TAA GGC ACG GGT AGC AC	GTA CTT CTG TGA CAC CGG CA
*β-Actin*	CAT CCG TAA AGA CCT CTA TGC CAA C	ATG GAG CCA CCG ATC CAC A

### Western blot analysis

Proteins were extracted from cells or spinal cords using radio immunoprecipitation assay lysis buffer (Beyotime, Shanghai, China) containing phenylmethanesulfonyl fluoride (Beyotime). The samples were centrifuged at 12,000 × *g* for 15 minutes at 4°C, and the protein concentration in the supernatant was determined using bicinchoninic acid (Beyotime). The proteins were separated by sodium dodecyl sulfate poly acrylamide gel electrophoresis and then transferred to polyvinylidene difluoride membranes. The membranes were blocked in phosphate-buffered saline with Tween-20 containing 5% bovine serum albumin (Solarbio, Beijing, China) at room temperature (20–25°C) for 1.5 hours. The membranes were then incubated with the following primary antibodies at 4°C overnight: rabbit anti-LC3 (1:1000, Proteintech, Wuhan, China, Cat# 14600-1-AP, RRID: AB_2137737), rabbit anti-PINK1 (1:1000, Proteintech, Cat# 23274-1-AP, RRID: AB_2879244), rabbit anti-occludin (1:1000, Proteintech, Cat# 66378-1-Ig, RRID: AB_2881755), rabbit anti-TOMM20 (1:1000, Proteintech, Cat# 66777-1-Ig, RRID: AB_2882123), rabbit anti-β-actin (1:1000, Proteintech, Cat# 20536-1-AP, RRID: AB_10700003), mouse anti-parkin (1:1000, Abcam, Cambridge, MA, USA, Cat# ab77924, RRID: GR3318878-9), mouse anti-ZO-1 (1:1000, Invitrogen, Carlsbad, CA, USA, Cat# 33-9100, RRID: AB_2533147), and rabbit anti-claudin-5 (1:1000, Invitrogen, Cat# 35-2500, RRID: AB_2533200). After washing with Tris-buffered saline with Tween 20, the membranes were incubated with the following horseradish peroxidase (HRP)-conjugated secondary antibodies for 1 hour at room temperature: goat anti-rabbit HRP (1:3000, InCellGene, Cimarron Path San Antonio, TX, USA, Cat# SA10011, RRID: 5551226088) and goat anti-mouse HRP (1:3000, InCellGene, Cat# SA10010, RRID: 5551226286). The protein bands were visualized using an enhanced chemiluminescence (ECL) detection reagent (Millipore, Billerica, MA, USA). A GE Healthcare Amersham Imager 600 Station (GE Healthcare, Stockholm, Sweden) was used for imaging. Target protein expression was quantified using ImageJ software and normalized to that of β-actin.

### Immunofluorescence staining

Spinal cord tissue from the T9 level was dehydrated with 4% PFA and 30% sucrose, embedded in optimal cutting temperature compound (Sakura, Torrance, CA, USA), frozen, and sectioned into 7-μm slices using a freezing microtome (CM1900, Leica Microsystems). Cultured cells were washed three times for 5 minutes each in phosphate-buffered saline and then incubated in 0.3% Triton X-100 for 20 minutes. Both frozen sections and cultured cells were blocked with 5% bovine serum albumin for 1 hour, followed by overnight incubation with the following primary antibodies at 4°C: rat anti-CD31 (1:200, R&D Systems, Minneapolis, MN, USA, Cat# AF-3628, RRID: 9259767), rabbit anti-LC3 (1:200, Proteintech, Cat# 14600-1-AP, RRID: AB_2137737), rabbit anti-PINK1 (1:200), rabbit anti-TOMM20 (1:200), mouse anti-ZO-1 (1:250), rabbit anti-Claudin-5 (1:200), mouse anti-Tuj1 (1:150, Abcam, Cat# ab7751, RRID: GR309497-9), and mouse anti-NeuN (1:200, Abcam, Cat# ab104224, RRID: GR3456813-1). The next day, the slides were washed with phosphate-buffered saline and incubated with the following secondary antibodies for 1 hour at room temperature: Alexa Fluor 488 goat anti-mouse (1:500, Jackson ImmunoResearch, West Grove, PA, USA, Cat# 115-545-003, RRID: AB_2338840), Alexa Fluor 594 goat anti-rabbit (1:300, Jackson ImmunoResearch, Cat# 111-585-003, RRID: AB_2338059), and Alexa Fluor 488 goat anti-rat (1:300, Abcam, Cat# ab150157, RRID: GR3444870-1). After phosphate-buffered saline washes, 4′,6-diamidino-2-phenylindole staining was performed. Imaging was conducted using a fluorescence microscope (BX51, Olympus, Tokyo, Japan).

### MitoTracker and LysoTracker staining

bEnd.3 cells were inoculated into six-well cell culture plates. When the cell confluence reached 70%–80%, the cells were subjected to OGD and treated with PBM. The live cells were directly stained with MitoTracker Red CMXRos (Cat# C1035, Beyotime) or LysoTracker Green probes (Cat# C1047, Beyotime) according to the manufacturer’s instructions and imaged using a fluorescence microscope (BX51, Olympus).

### Measurement of mitochondrial membrane potential

Mitochondrial membrane potential (ΔΨm) was measured using a commercial assay kit. Briefly, bEnd.3 cells were incubated with JC-1 staining solution (Cat# C2006, Beyotime) at 37°C for 20 minutes, washed with JC-1 staining buffer, and immersed in culture medium. Images of positively stained cells were captured using a fluorescence microscope. Normal mitochondria produced red fluorescence, while depolarized or inactive mitochondria produced green fluorescence. ΔΨm was calculated as the ratio of red to green fluorescence.

### Assessment of oxidative stress

bEnd.3 cells were cultured in a 24-well plate and then incubated with 2,7 dichlorofluorescein diacetate (Beyotime) in the absence of light for 20 minutes. Next, the cells were washed three times with serum-free medium. Reactive oxygen species (ROS) generation was evaluated by measuring dichlorofluorescein fluorescence with a fluorescence microscope, using an excitation wavelength of 488 nm and an emission wavelength of 519 nm. The fluorescence intensity values were then normalized to those obtained from the untreated control group.

### Statistical analysis

No statistical methods were used to predetermine sample size. The experiments were repeated at least three times for each group, and the evaluators were blinded to the group assignments. Data are shown as mean ± standard deviation (SD), with each experiment repeated at least three times. Statistical analysis was performed used GraphPad Prism (version 9.5.0 for Windows, GraphPad Software, Boston, MA, USA, www.graphpad.com). Unpaired two-tailed Student’s *t*-test was used to compare two groups, while comparisons among more than two groups were performed by one-way analysis of variance followed by Tukey’s *post hoc* test. Two-way repeated measure analysis of variance, followed by Tukey’s *post hoc* test, was used to examine differences between groups at different times. *P* < 0.05 was considered statistically significant.

## Results

### Photobiomodulation restores blood–spinal cord barrier integrity and promotes spinal cord injury repair

We used EB detection to evaluate the effect of PBM on BSCB permeability from 1 to 14 dpi. As depicted in **Figure 1A–C**, EB extravasation reached its peak on day 1 post-injury and showed no significant difference from the control group on day 14. PBM significantly reduced EB extravasation at the injury site at 1, 3, 7, and 14 dpi (**Figure 1C**). BMS scoring of mice in the Sham group, SCI group, and SCI + PBM group before surgery and on days 1, 3, 7, 14 and 28 after surgery showed that motor function deteriorated significantly after SCI (**Figure 1D**). However, compared with the SCI group, mice in the SCI + PBM group showed significant improvement in motor dysfunction from day 7 to day 28 after injury (**Figure 1D**). Louisville Swim Scale scoring yielded similar results: from days 14 to 28 after injury, the SCI + PBM mice exhibited greater motor function recovery than the SCI mice (**Figure 1E**). Footprint analysis 14 days after injury (**Figure 1F**) showed that the stride length of mice in the SCI + PBM group was significantly greater than that in the SCI group (**Figure 1G**), and the motor function of the hindlimbs was clearly improved. Motor-evoked potential analysis performed 28 days following SCI, showed that, in contrast to the SCI group, the amplitude was significantly augmented and the latency was significantly reduced in mice in the SCI + PBM group, suggesting superior neurological function recovery (**Figure 1H** and **I**). The therapeutic effect of PBM on neural repair after SCI was analyzed by immunofluorescence staining of NeuN^+^ neurons and Tuj1^+^ axons on days 14 and 28 after injury, respectively. On the 14^th^ day after SCI, there were more NeuN^+^ neurons in the SCI area of mice in the SCI + PBM group than in the SCI group (**Figure 1J–L**). Consistent with the functional evaluation results, we observed more Tuj1^+^ axons in the SCI area of mice in the PBM group 28 days after SCI compared with the SCI group (**Figure 1M–O**). In summary, PBM restored BSCB integrity and facilitated neuronal regeneration, thereby enhancing hindlimb motor function recovery following SCI.

### Photobiomodulation regulates angiogenesis, endothelial cell adhesion, and tight junction function after spinal cord injury

**Figure 2:**
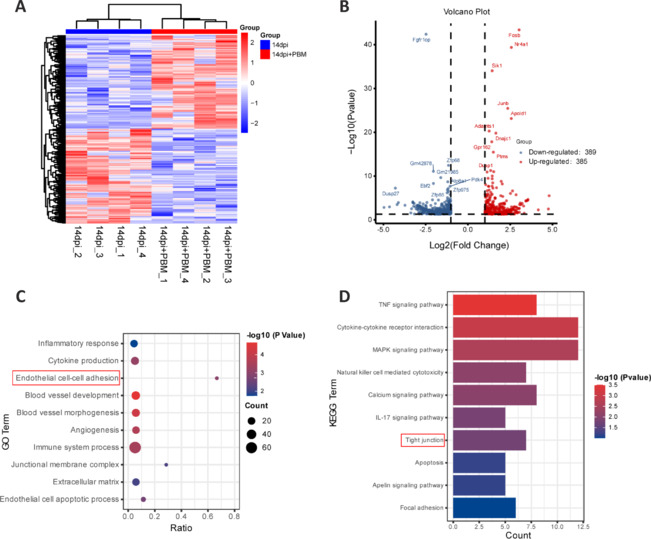
PBM regulates angiogenesis, endothelial cell adhesion, and tight junction function after spinal cord injury. RNA transcriptome sequencing was performed on mouse spinal cord tissues from the injury-only group (14 dpi) and the injured and PBM-treated (14 dpi + PBM) group on day 14 post-injury. (A) Heatmap of DEGs between the two groups of spinal cord tissues (|log2FoldChange| ≥ 1, *P*-value < 0.05). (B) Volcano plot of DEGs between the two groups. Red and blue dots represent up- and downregulated DEGs, respectively. (C) GO analysis of DEGs between the groups highlighted functions related to the inflammatory response and the blood–spinal cord barrier. (D) KEGG enrichment analysis of DEGs among the groups (TOP10). Red boxes indicate enriched functions and pathways closely related to BSCB integrity. BSCB: Blood–spinal cord barrier; DEG: differentially expressed gene; GO: Gene Ontology; KEGG: Kyoto Encyclopedia of Genes and Genomes; SCI: Spinal cord injury.

Given our observation that PBM promoted BSCB integrity and neural regeneration in mice with SCI, we next performed RNA transcriptome sequencing of the spinal cord to determine the underlying molecular mechanisms of these effect. Spinal cord tissue samples measuring 0.8 cm in length were collected from the center of the injury site from mice in both the SCI group and the SCI + PBM group (*n* = 4). As shown in heatmap and volcano plot in **Figure 2A** and **B**, 385 DEGs were upregulated and 389 DEGs were downregulated in the SCI + PBM group (14 dpi + PBM) compared with the SCI group (14 dpi). Consistent with our previous studies and earlier observations from the current study, GO analysis of the DEGs showed that PBM significantly promotes angiogenesis, reduces endothelial cell apoptosis, enhances endothelial cell adhesion, and inhibits secondary neuroinflammation after SCI (**Figure 2C**). KEGG analysis of the DEGs suggested that PBM also regulates intercellular TJs (**Figure 2D**). Given the central role of the BSCB, which is dominated by vascular endothelial cells, in inflammatory cell infiltration and secondary neuroinflammation after SCI, these results suggest that PBM modulates BSCB integrity after SCI by promoting vascular regeneration and vascular endothelial cell intercellular adhesion via regulation of intercellular TJ proteins.

### Photobiomodulation promotes the expression of proteins that form tight junction between the endothelial cells that comprise the blood–spinal cord barrier

**Figure 3:**
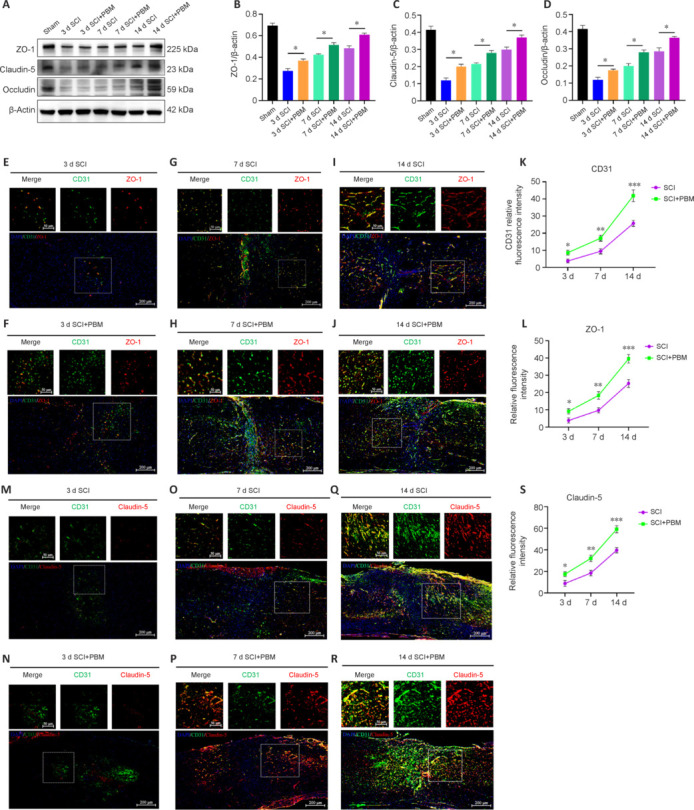
PBM promotes the expression of tight junction proteins between endothelial cells in the blood–spinal cord barrier. (A) Representative western blot images of expression of the tight junction proteins ZO-1, Occludin, and Claudin-5 in spinal cord tissues from mice in the Sham group and the SCI and SCI + PBM groups at 3, 7, and 14 dpi. (B–D) Quantitation of expression of the tight junction proteins ZO-1, Claudin-5, and Occludin in each group (*n* = 6 animals per group). (E–J) Representative immunofluorescence images of ZO-1 (red, Alexa Fluor 594) and CD31 (green, Alexa Fluor 488) in the spinal cord tissues of mice in the SCI and SCI + PBM groups at 3, 7, and 14 dpi (*n* = 6 animals per group). The selected regions are displayed at a higher magnification. Scale bars: 200 μm, 50 μm (higher magnification). (K, L) Quantification of CD31^+^ and ZO-1^+^ fluorescence intensity (*n* = 6 animals per group at each time point). (M–R) Representative immunofluorescence images of Claudin-5 (red, Alexa Fluor 594) and CD31 (green, Alexa Fluor 488) in the spinal cord of SCI and SCI + PBM mice at 3, 7, and 14 dpi (*n* = 6 animals per group). The selected regions are displayed at a higher magnification. Scale bars: 200 μm, 50 μm (higher magnification). (S) Quantification of CD31 expression (*n* = 6 animals per group). Data are shown as means ± SDs. *P < 0.05, ** *P* < 0.01, *** *P* < 0.001 (one-way analysis of variance followed by Tukey’s *post hoc* test (B–D) or two-way repeated measure analysis of variance followed by Tukey’s *post hoc* test (K, L, S). dpi: Day(s) post-injury; PBM: photobiomodulation; SCI: spinal cord injury.

TJ proteins are crucial for maintaining BSCB integrity, and their expression levels are positively correlated with BSCB integrity (Zhu et al., 2023). Therefore, we detected TJ protein expression in injured spinal cord tissues from mice in the SCI + PBM group and SCI group by immunoblotting at 3, 7, and 14 dpi. TJ protein expression was significantly lower in the SCI group compared with the Sham group, while treatment with PBM increased TJ protein expression (**Figure 3A–D**). Furthermore, immunofluorescence staining of mouse spinal cord tissues at 3, 7, and 14 dpi showed that the density of CD31^+^ blood vessels at the injury site was significantly higher in mice in the SCI + PBM group compared with mice in the SCI group (**Figure 3E–K**). Furthermore, expression of the TJ protein ZO-1 (**Figure 3E–L**) correlated with blood vessel density in both groups. Moreover, PBM treatment promoted expression of the TJ protein Claudin-5 in CD31^+^ vascular endothelial cells in the spinal cord on days 3, 7, and 14 after SCI (**Figure 3M–S**). Additionally, CD31^+^ vascular endothelial cells at the site of SCI exhibited strong colocalization with both ZO-1 and Claudin-5 (**Figure 3E–S**).

### Photobiomodulation enhances microvascular endothelial cell tight junction protein expression

**Figure 4:**
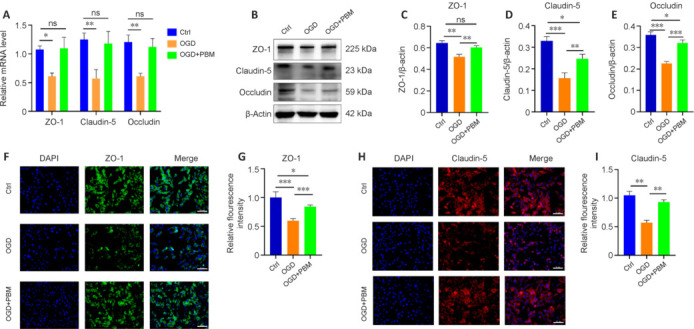
PBM enhances tight junction protein expression in microvascular endothelial cells. (A) Real-time PCR analysis of the expression of the tight junction proteins ZO-1, Occludin and Claudin-5 in bEnd.3 cells in the Ctrl, OGD, and OGD + PBM groups (*n* = 3 samples per group). (B) Western blot analysis of the expression of ZO-1, Occludin, and Claudin-5 in bEnd.3 cells in the Ctrl, OGD, and OGD + PBM groups (*n* = 3 samples per group). (C–E) Quantification of ZO-1, Claudin-5, and Occludin expression. (F, H) Immunofluorescence staining for the tight junction proteins ZO-1 (green, Alexa Fluor 488) and Claudin-5 (red, Alexa Fluor 594) in bEnd.3 cells in the Ctrl, OGD, and OGD + PBM groups (*n* = 4 samples per group). Scale bars: 50 μm. (G, I) Quantification of ZO-1 and Claudin-5 fluorescence intensity. Data are shown as mean ± SD. *P < 0.05, **P < 0.01, ***P < 0.001 (one-way analysis of variance followed by Tukey’s *post hoc* test). Ctrl: Control; DAPI: 4′,6-diamidino-2-phenylindole; OGD: oxygen-glucose deprivation; PBM: photobiomodulation; PCR: polymerase chain reaction.

To gain a more comprehensive understanding of the mechanism by which PBM affects TJs, we subjected bEnd.3 cells to OGD *in vitro* and treated them with PBM. Oxidative stress induced by OGD led to a decrease in the mRNA and protein levels of TJ proteins (ZO-1 and Claudin-5) in endothelial cells, and this effect was rescued by PBM treatment (**Figure 4A–E**). Subsequently, we performed immunofluorescence staining for ZO-1 and Claudin-5 and found that the fluorescence intensity of ZO-1 (**Figure 4F–G**) and Claudin-5 (**Figure 4H–I**) in the PBM treatment group was higher than that in the OGD group. These findings indicate that PBM mitigates OGD-induced injury in brain microvascular endothelial cells and enhances the expression of intercellular TJ proteins. This observation aligns with our earlier *in vivo* results showing that PBM modulates vascular endothelial cells and their associated TJ proteins within the BSCB following SCI.

### Photobiomodulation reduces oxidative stress and mitochondrial damage in endothelial cells

**Figure 5:**
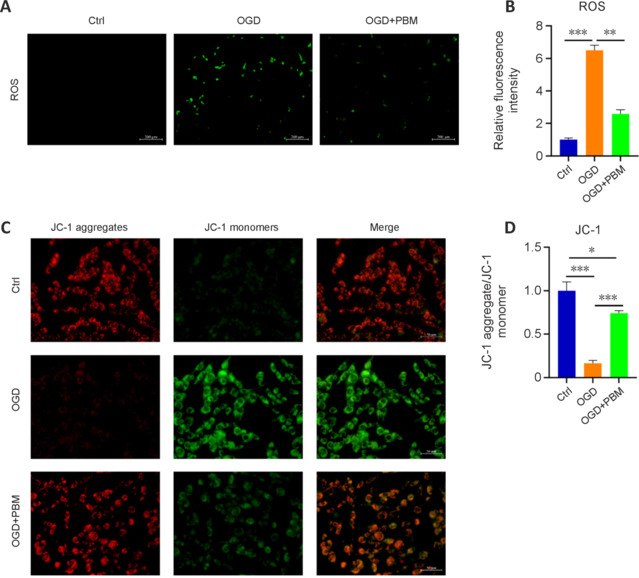
PBM reduces oxidative stress and mitochondrial damage in bEnd.3 cells subjected to OGD. (A) Dichlorodihydrofluorescein diacetate probe detection of ROS production in bEnd.3 cells from the Ctrl, OGD, and OGD + PBM groups. PBM treatment inhibited OGD-induced ROS generation. Scale bars: 200 μm. (B) Quantification of ROS fluorescence intensity (*n* = 3 samples per group). (C) JC-1 probe detection of the mitochondrial membrane potential of bEnd.3 cells from the Ctrl, OGD, and OGD + PBM groups. Compared with the OGD group, PBM treatment resulted in enhanced JC-1 red fluorescence intensity indicating normal mitochondria and reduced JC-1 green fluorescence intensity indicating damaged mitochondria. Scale bars: 50 μm. (D) Ratio of JC-1 red to JC-1 green fluorescence (*n* = 3 samples per group). Data are shown as mean ± SD. *P < 0.05, **P < 0.01, ***P < 0.001 (one-way analysis of variance followed by Tukey’s *post hoc* test). Ctrl: Control; DAPI: 4′,6-diamidino-2-phenylindole; OGD: oxygen-glucose deprivation; PBM: photobiomodulation; ROS: reactive oxygen species.

To elucidate the mechanism by which PBM enhances TJ protein expression, we analyzed mitochondrial function and oxidative stress levels in vascular endothelial cells, which are the primary targets of PBM. OGD increased ROS production, and this effect was inhibited by PBM treatment (**Figure 5A** and **B**). Furthermore, JC-1 fluorescence analysis of ΔΨm showed that PBM treatment enhanced the red fluorescence intensity of JC-1 labeling, indicating normal mitochondria, and reduced the green fluorescence intensity of JC-1 labeling, indicating damaged mitochondria, compared with the OGD group (**Figure 5C** and **D**). This suggests that PBM effectively mitigates ΔΨm decline in compromised vascular endothelial cells, inhibits ROS production, and facilitates the recovery of damaged mitochondria within these cells.

### Photobiomodulation promotes mitophagy in endothelial cells through the PINK1/Parkin pathway

Oxidative stress induces mitochondrial damage in endothelial cells, impairing mitochondrial function and triggering mitophagy (Shefa et al., 2019). Mitophagy is crucial for preserving the health of vascular endothelial cells and maintaining BSCB integrity because it prevents endothelial cell apoptosis mediated by the mitochondrial pathway and degradation of intercellular TJ proteins (Uoselis et al., 2023). Therefore, we next asked whether PBM mitigates oxidative stress–induced injury and TJ protein degradation in endothelial cells by enhancing mitophagy.

**Figure 6:**
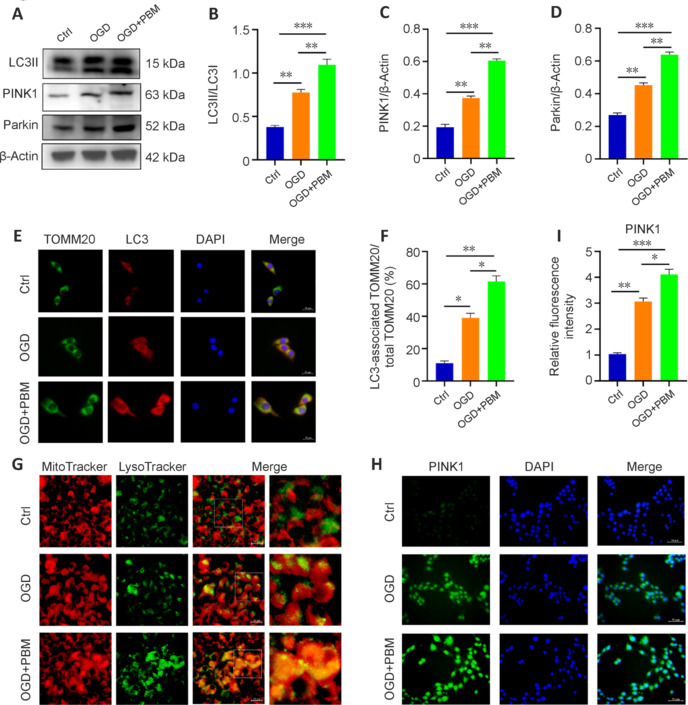
PBM promotes mitophagy in endothelial cells through the PINK1/Parkin pathway. (A) Western blot detection of LC3, PINK1, and Parkin expression in bEnd.3 cells from the Ctrl, OGD, and OGD + PBM groups. (B–D) Quantitative analysis of LC3II/I, PINK1, and Parkin expression (*n* = 3 samples per group). (E) Immunofluorescence staining of the mitochondrial membrane protein marker TOMM20 (green, Alexa Fluor 488) and the autophagy marker LC3 (red, Alexa Fluor 594) in bEnd.3 cells from the Ctrl, OGD, and OGD + PBM groups (*n* = 3 samples per group). Colocalization of LC3 and TOMM20 was more pronounced in the PBM-treated group compared with the OGD group. Scale bars: 20 μm. (F) Quantification of the ratio of LC3-associated TOMM20 to total TOMM20. (G) MitoTracker Red and LysoTracker Green staining were used to detect the colocalization of mitochondria and lysosomes in bEnd.3 cells from each group (*n* = 3 samples per group). Colocalization of mitochondria and lysosomes was significantly increased after OGD injury, and PBM treatment further enhanced this colocalization. Scale bars: 20 μm. (H) Representative fluorescence images of PINK1 (green, Alexa Fluor 488) in bEnd.3 cells. PINK1/Parkin-related autophagy of endothelial cells in the PBM group was stronger than that in OGD group. Scale bars: 50 μm. (I) Fluorescence quantification of PINK1 (*n* = 3 samples per group). Data are shown as mean ± SD. *P < 0.05, **P < 0.01, ***P < 0.001 (one-way analysis of variance followed by Tukey’s *post hoc* test). Ctrl: Control; DAPI: 4′,6-diamidino-2-phenylindole; OGD: oxygen-glucose deprivation; PBM: photobiomodulation; PINK1: PTEN-induced kinase 1.

Microtubule-associated protein light chain 3 (LC3) is an autophagosome marker (Li et al., 2022b). We observed elevated expression of the mitophagy indicators LC3II/I, PINK1, and Parkin in endothelial cells subjected to OGD, and treatment with PBM enhanced these effects (**Figure 6A–D**). TOMM20 is located on the mitochondrial membrane. LC3 and TOMM20 play important roles in mitophagy, and changes in their expression and localization reflect mitophagy activity (Liu et al., 2022). Immunostaining showed LC3 colocalization with TOMM20 (**Figure 6E** and **F**) that increased following OGD and became even more pronounced with PBM treatment. Autophagosomes encapsulate and transport damaged mitochondria to lysosomes (Kong et al., 2019). When we labeled mitochondria with MitoTracker Red and lysosomes with LysoTracker, we observed a significant increase in mitochondria and lysosome colocalization post-OGD and found that PBM treatment further augmented this colocalization (**Figure 6G**), indicating increased mitophagy. Collectively, these findings indicate that PBM promotes mitophagy in endothelial cells. The mitochondrial kinase PINK1 and the E3 ubiquitin ligase Parkin are pivotal proteins involved in mitophagy regulation (Quinn et al., 2020). PINK1 accumulates on impaired mitochondria and recruits Parkin from the cytosol, thereby initiating mitophagy (Kane et al., 2014). To determine whether PBM activates mitophagy via the PINK1/Parkin pathway, we quantified PINK1 and Parkin expression in bEnd.3 cells. Western blot analysis demonstrated a marked increase in PINK1 and Parkin expression in the OGD group compared with the Ctrl group, with a further increase induced by PBM treatment (**Figure 6A** and **C**). Immunofluorescence staining and immunoblotting yielded similar results. These observations substantiate the involvement of PINK1/Parkin signaling in the PBM-induced augmentation of mitophagy (**Figure 6H** and **I**).

### Inhibiting mitophagy increases reactive oxygen species accumulation in endothelial cells, reversing the therapeutic effects of photobiomodulation

**Figure 7:**
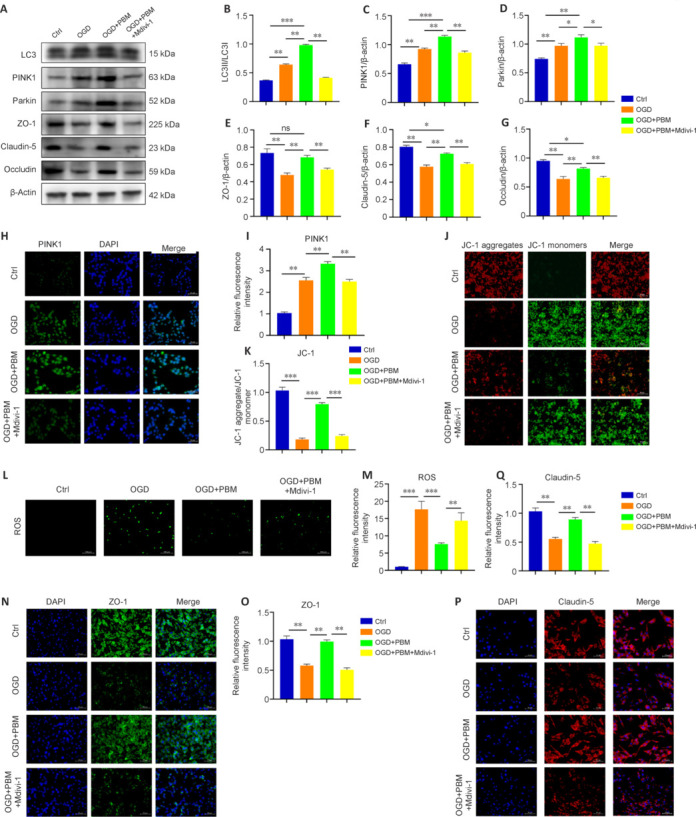
Inhibition of mitophagy results in increased ROS accumulation in endothelial cells and reverses the therapeutic effects of PBM. (A) Immunoblotting analysis of LC3, PINK1, Parkin, and TJ protein (ZO-1, Claudin-5, and Occludin) expression in bEnd.3 cells from the Ctrl, OGD, OGD + PBM, and OGD + PBM + Mdivi-1 groups. (B–G) Quantitative analysis of LC3, PINK1, Parkin, and TJ protein (ZO-1, Claudin-5, and Occludin) expression in each group of cells (*n* = 3 samples per group). (H) Immunofluorescence staining of PINK1 (green, Alexa Fluor 488) in cells from each group. Pretreatment with Mdivi-1 attenuated the PBM-induced increase in expression of the mitophagy-related proteins PINK1 and Parkin, as evidenced by a decrease in the LC3-II/I ratio. Scale bars: 50 μm. (I) Quantification of PINK1 fluorescence intensity in the OGD, OGD + PBM, and OGD + PBM + Mdivi-1 groups relative to the control group (*n* = 3 samples per group). (J) JC-1 probe detection of the mitochondrial membrane potential of bEnd.3 cells in each group. Inhibition of mitophagy by Mdivi-1 counteracted the PBM-induced decrease in ΔΨm. Scale bars: 50 μm. (K) Ratio of JC-1 red to JC-1 green fluorescence (*n* = 3 samples per group). (L) Dichlorodihydrofluorescein diacetate probe detection of ROS production in each group of bEnd.3 cells. Mdivi-1 treatment increased ROS levels in endothelial cells. (M) Quantification of ROS fluorescence intensity (*n* = 3 samples per group). (N) Representative immunofluorescence images of ZO-1 (green, Alexa Fluor 488) in bEnd.3 cells from each group. Administration of Mdivi-1 blocked the PBM-induced increase in ZO-1 and Claudin-5 expression. Scale bars: 50 μm. (O) Quantification of ZO-1 fluorescence (*n* = 3 samples per group). (P) Representative immunofluorescence images of Claudin-5 (red, Alexa Fluor 594) in bEnd.3 cells from each group. Pretreatment with Mdivi-1, a mitophagy inhibitor, blocked the PBM-induced increased in expression of the tight junction proteins ZO-1 and Claudin-5. Scale bars: 50 μm. (Q) Quantification of Claudin-5 fluorescence. (*n* = 3 samples per group). Data are shown as mean ± SD. *P < 0.05, **P < 0.01, ***P < 0.001 (one-way analysis of variance followed by Tukey’s *post hoc* test). Ctrl: Control; DAPI: 4′,6-diamidino-2-phenylindole; OGD: oxygen-glucose deprivation; PBM: photobiomodulation; PINK1: PTEN-induced kinase 1.

Next, we treated endothelial cells with Mdivi-1, a specific inhibitor of mitophagy, to determine whether PBM exerts is protective effects through mitophagy. Western blot analysis showed that pretreatment with Mdivi-1 attenuated the PBM-induced enhancement of mitophagy, as evidenced by a reduction in the LC3-II/I ratio (**Figure 7A** and **B**). Expressions of the mitophagy-related proteins PINK1 and Parkin were restored to levels comparable to the control (**Figure 7A–D**). To evaluate the impact of Mdivi-1 treatment on the BSCB, we quantified the expression of TJ proteins in bEnd.3 cells by western blot and found that the PBM-induced upregulation of TJ proteins (ZO-1, Claudin-5, and Occludin) was reversed following Mdivi-1 treatment (**Figure 7A** and **E–G**). PINK1 immunofluorescence staining yielded similar results (**Figure 7H** and **I**), suggesting effective inhibition of mitophagy. JC-1 fluorescence probe analysis indicated that inhibition of mitophagy by Mdivi-1 counteracted the PBM-induced reduction in ΔΨm and mitigated mitochondrial damage (**Figure 7J** and **K**). Subsequently, we examined the impact of Mdivi-1 on oxidative stress and found that Mdivi-1 treatment resulted in elevated ROS levels (**Figure 7L** and **M**). This increase in ROS may be attributable to decreased clearance of damaged mitochondria following inhibition of mitophagy, thereby leading to accumulation of the ROS released by the damaged mitochondria.

Immunofluorescence staining showed that Mdivi-1 treatment inhibited the PBM-induced increase in the expression of the TJ proteins ZO-1 and Claudin-5 (**Figure 7N–Q**). These findings indicate that PBM protects endothelial cells via mitophagy.

## Discussion

After SCI, the injury site rapidly attracts various immune cells such as macrophages (Li et al., 2022a). We previously demonstrated that PBM can induce macrophages to transition from a pro-inflammatory phenotype to an anti-inflammatory phenotype through multiple signaling pathways, thus accelerating SCI repair (Ma et al., 2022; Ju et al., 2023). Given the key role of macrophages in inflammation and tissue repair, as well as their potentially close connection with BSCB function, in this study we assessed the effect of PBM on the BSCB. By evaluating EB extravasation *in vivo*, we confirmed that PBM promotes recovery of BSCB integrity after SCI. Assessment of motor function and nerve regeneration showed that PBM promoted motor function recovery in mice. *In vitro*, PBM treatment bEnd.3 subjected to OGD reduced ROS production, promoted recovery of mitochondrial function, and enhanced PINK1/Parkin-mediated mitophagy. Treatment with the mitophagy-specific inhibitor Mdivi-1 resulted in a significant increase in ROS levels, reversing the PBM-induced increase in TJ protein expression. These findings illustrate that PBM reduces oxidative stress and promotes BSCB recovery by enhancing mitophagy. This evidence sheds new light on the potential of PBM therapy for SCI.

BSCB protects the spinal cord tissue and ensures normal physiological functioning of nerve cells by strictly controlling substance exchange, maintaining the stability of the internal environment, achieving immune isolation, and protecting nerve function (Lin et al., 2024; Xiong et al., 2024). However, SCI can endanger the BSCB by damaging its structure, triggering inflammation, interfering with endothelial cell function, causing oxidative stress imbalance, and hindering self-repair (Zhou et al., 2023; Wang et al., 2024). Jiang et al. (2023) examined BSCB permeability after SCI in mice and reported that EB leakage peaked at 1 day, while BSCB function was re-established by 14 days after injury. In our study, we systematically investigated changes in mouse BSCB permeability following SCI. Consistent with previous reports, our results indicate maximal BSCB disruption at 1 day post-SCI, with partial recovery by day 14 post-injury. Few studies have reported on the effects of PBM on the BSCB. In this study, we carefully examined the effects of PBM on BSCB following SCI. Notably, PBM treatment accelerated BSCB recovery.

We further studied TJs post-SCI, as TJs play a crucial role in maintaining blood–brain barrier and BSCB function (Yang et al., 2019; Xie et al., 2023). The downregulation of TJ protein (ZO-1, occludin, and claudin-5) expression that we observed suggests TJ disruption post-SCI. PBM treatment significantly promoted TJ protein expression post-SCI. A previous study showed that PBM can promote early angiogenesis and accelerate muscle and nerve reconstruction, thereby facilitating recovery from SCI (Tobelem et al., 2023). In our study, we found that PBM enhanced expression of the vascular marker CD31, suggesting that it had a pro-angiogenic effect.

Mitochondria play a crucial role in ROS production, and excessive ROS generation can result in mitochondrial damage, further exacerbating ROS production (Zorov et al., 2014; Kuznetsov et al., 2022). The oxidative stress that occurs after SCI generates a large amount of ROS, leading to disruption of the oxidation/antioxidation system in the body (Yin et al., 2024). ROS can directly attack TJ proteins between endothelial cells in the BSCB, and the damage induced to these structures significantly increases BSCB permeability (van Doorn et al., 2012; Jung et al., 2016). This enables harmful substances in the blood to more easily invade the spinal cord tissue and disrupt its normal physiological environment. Excessive ROS levels can give rise to lipid peroxidation, protein oxidation, and DNA damage, influencing the survival, proliferation, and migration capabilities of endothelial cells (Wei et al., 2022). Furthermore, excessive ROS generation can injure the mitochondrial membrane, affect the function of the mitochondrial respiratory chain, and cause a reduction in ATP production and an imbalance in cellular energy metabolism (Shi et al., 2022). This not only impacts the normal physiological activities of endothelial cells but also may trigger the mitochondrial-mediated apoptotic pathway, accelerating endothelial cell death (Kim et al., 2018). Therefore, anti-oxidative stress is an important target for treating BSCB damage after SCI. PBM is an emerging approach for treating oxidative stress. We previously reported that PBM can reduce oxidative stress after SCI in rats through multiple pathways (Zhu et al., 2022; Zhang et al., 2023). In this study, we found that PBM significantly reduced ROS production in mouse microvascular endothelial cells, reduced mitochondrial damage, and enhanced the antioxidant capacity of cells. Our results indicate that PBM protects the BSCB by reducing oxidative stress.

Mitophagy was a main focus of our exploration of the mechanism by which PBM reduces oxidative stress and promotes BSCB recovery. When cells encounter stress and damage, mitophagy is activated to selectively eliminate damaged mitochondria (Shefa et al., 2019). This process helps maintain mitochondrial quality and function within the cells, reduces the generation of harmful ROS, and inhibits excessive activation of the inflammatory response (Swerdlow and Wilkins, 2020). Xu et al. (2023) reported that Schwann cell exosomes enhance mitophagy, reducing the production of ROS and inflammatory cytokines induced by injury. Mao et al. (2022) reported that maltol treatment enhances PINK1/Parkin-mediated mitophagy in PC12 cells, reducing oxidative stress and promoting recovery of mitochondrial function. The nature of the relationship between PBM and mitophagy is unclear, but emerging evidence suggests that they are closely associated. Previous studies have demonstrated that PBM targets mitochondria, regulating mitochondrial damage and restoring mitochondrial function in various cells (Zhu et al., 2022; Zhang et al., 2023). In addition, PBM can enhance PINK1 and Parkin expression in adipose-derived stem cells from patients with diabetes (Fallahi et al., 2023). Therefore, we hypothesized that the therapeutic effects of PBM on BSCB damage are mediated by mitophagy. To test this, we evaluated the effect of PBM on mitophagy in endothelial cells after oxidative stress. When autophagy occurs, LC3 gradually converts to LC3II and localizes to the surface of autophagosomes, marking autophagic vesicles for delivery to lysosomes, where they are degraded (Porter et al., 2013). We found that mitophagy in bEnd.3 cells increased significantly after PBM treatment, as indicated by colocalization of mitochondria and lysosomes, as well as increased expression of LC3II. Furthermore, immunofluorescence showed that PINK1 and Parkin expression increased after oxidative stress. These results suggests that PBM promotes mitophagy through the classical PINK1/Parkin signaling pathway.

The question remains, however, whether PBM alleviates oxidative stress by stimulating mitophagy in endothelial cells. Mdivi-1 is an inhibitor of mitochondrial division that inhibits mitophagy. Therefore, we used Mdivi-1 to investigate the specific role of mitophagy in PBM-mediated protection of the BSCB. Treatment with the mitophagy inhibitor prevented the decrease in ROS levels and the decline in ΔΨm. Additionally, the use of the mitophagy inhibitor inhibited the upregulation of TJ protein expression induced by PBM. Immunofluorescence further confirmed that treatment with Mdivi-1 weakened the protective effect of PBM on the BSCB. These results indicate that inhibiting mitophagy can reduce PBM’s antioxidant effect and protection of the BSCB. However, enhancing mitophagy may also promote BSCB repair by regulating cellular energy metabolism and cell survival signaling pathways, which helps restore TJs between endothelial cells, reduces BSCB permeability, and ultimately restores normal physiological function of the BSCB.

Although our study identified an important connection between PBM-mediated promotion of mitophagy and BSCB repair, there are still many answered questions that require further exploration. For example, the precise molecular mechanism by which PBM regulates mitophagy-related pathways remains unclear. In addition, whether there are differences in PBM efficacy in different degrees and types of SCIs, and how to optimize PBM parameters to achieve the greatest therapeutic effect, require more in-depth study.

The present study had some limitations. Our findings suggest that PBM can reduce ROS production after SCI and attenuate BSCB injury through mitophagy. However, we did not thoroughly investigate the specific mechanisms by which PBM promotes the restoration of BSCB integrity after SCI. Second, we only examined the effects of PBM on the BSCB in mice during the acute and subacute phases (up to 14 days) of injury, and subsequent studies are needed to investigate the effects of longer irradiation durations. In addition, we included only male mice in this study, and additional data on females and mice of different ages are needed. In conclusion, our findings suggest that PBM can reduce ROS production after SCI and alleviate BSCB damage through mitophagy, thus promoting recovery from SCI. Our study provides new insights into the regulation of neuroinflammation and BSCB integrity by PBM after SCI and its related mechanisms, as well as novel ideas for SCI treatment.

## Additional file:

***Additional Figure 1:*** *Experimental timeline.*

Additional Figure 1 Experimental timeline.
The mice were randomly divided into the SCI group and the SCI + PBM group. At 0,1, 3, 7, 14 and 28 days after SCI, EB staining, immunofluorescence staining, BMS, Louisville Swim Scale, footprint analysis, transcriptome sequencing, neurophysiological assessments, and WB were used to elucidate the regulatory effects and related mechanisms of PBM on neural regeneration and BSCB integrity after SCI. BMS: Basso Mouse Scale; EB: Evans Blue; PBM: photobiomodulation; SCI: spinal cord injury; WB: Western blotting.

## Data Availability

*The data are available from the corresponding author on reasonable request*.
