# Supplementary figures and images for: Photobiomodulation repairs the blood–spinal cord barrier in a mouse model of spinal cord injury
Source: Neural Regen Res. 2025 Mar 25;21(6):2475–84. doi: 10.4103/NRR.NRR-D-24-01098 (PMC13211799; doi:10.4103/NRR.NRR-D-24-01098)

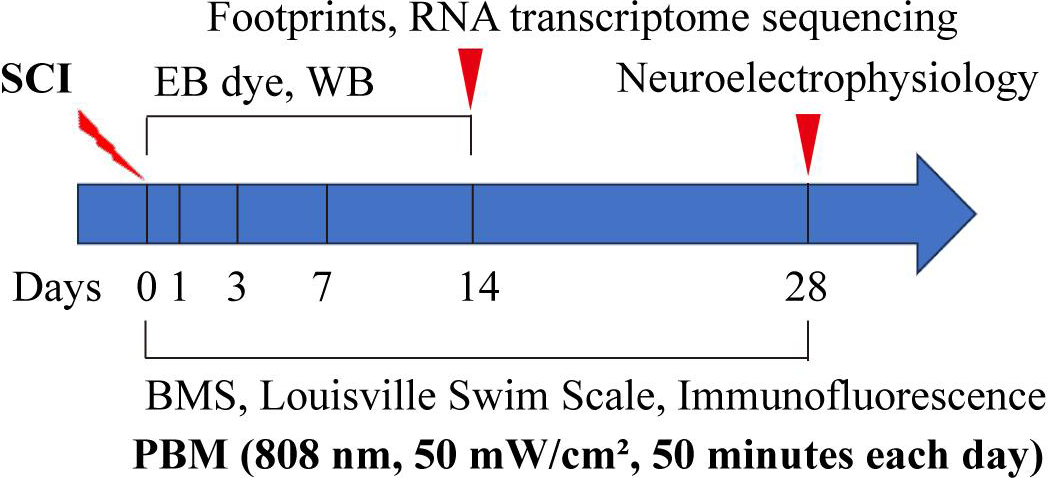

Supplement: Supplementary file 1 [file NRR-21-2475_Suppl1.tif]
